# Effectiveness of a brief psychological mindfulness-based intervention for the treatment of depression in primary care: study protocol for a randomized controlled clinical trial

**DOI:** 10.1186/s12888-019-2298-x

**Published:** 2019-10-16

**Authors:** Alba Lopez-Montoyo, Soledad Quero, Jesus Montero-Marin, Alberto Barcelo-Soler, Maria Beltran, Daniel Campos, Javier Garcia-Campayo

**Affiliations:** 1 Universitat Jaume I, Av. Vicente Sos Baynat s/n, 12006 Castellón, Spain; 2 CIBER de Fisiopatología de la Obesidad y Nutrición (CIBEROBN), Madrid, Spain; 3 Primary Care Prevention and Health Promotion Research Network (RedIAPP), Zaragoza, Spain; 4 University of Zaragoza, Zaragoza, Spain; 5 Aragón Health Research Institute (IIS Aragón), Zaragoza, Spain; 6 Department of Psychiatry, University of Zaragoza, Zaragoza, Spain; 7 Psychiatry Service, Miguel Servet Hospital, Zaragoza, Spain

**Keywords:** Depression, primary care, Mindfulness-based intervention, Low-intensity, Face-to-face, Internet delivered, Randomized controlled trial

## Abstract

**Background:**

Depressive symptoms are quite prevalent in Primary Care (PC) settings. The treatment as usual (TAU) in PC is pharmacotherapy, despite the high relapse rates it produces. Many patients would prefer psychotherapy, but specialized services are overloaded. Studies that apply Mindfulness-Based Interventions (MBIs) for the treatment of depression have obtained significant improvements. Brief low-intensity approaches delivered from PC could be a promising approach. This study aims to compare a low-intensity mindfulness intervention for the treatment of depression in PC using different intervention formats – a face-to-face MBI delivered in a group and the same MBI individually applied on the Internet – to a control group that will receive PC medical treatment as usual.

**Methods:**

A randomized controlled clinical trial will be conducted in PC, with about 120 depressed patients allocated (1:1:1) to three groups: “face-to-face MBI + TAU”, “Internet-delivered MBI + TAU”, and “TAU alone”. The MBI programs will be composed of four modules. The primary outcome will be depressive symptoms, measured through the Beck Depression Inventory, assessed at pre- and post-treatment and 6- and 12-month follow-ups. Other outcomes will be mindfulness, happiness, affectivity, quality of life, and the use of healthcare services. Intention-to-treat analysis using linear mixed models adjusted for baseline scores and routine sociodemographic analysis that could show baseline differences will be conducted. Per-protocol secondary outcome analyses will also be performed.

**Discussion:**

This is the first Spanish RCT to apply a low-intensity face-to-face MBI (plus TAU) to treat depression in PC settings compared to TAU (alone). Moreover, this study will also make it possible to evaluate the same MBI program (plus TAU), but Internet-delivered, considering their cost-effectiveness. Positive results from this RCT might have an important impact on mental health settings, helping to decrease the overload of the system and offering treatment alternatives beyond antidepressant medication through high-quality, flexible PC interventions.

**Trial registration:**

Clinical Trials.gov NCT03034343. Trial Registration date 24 January 2017, retrospectively registered.

## Background

Major Depression is a common mental disorder that produces great functional and social impairment all over the world. It is characterized by sustained states of negative affectivity and diminished positive affectivity. According to the World Health Organization (WHO), in 2020, this disorder will be the second most important cause of disability [1]. It has been estimated that around 14.0% of the European population have suffered from a lifetime history of major depressive disorder [2]. Specifically in Primary Care (PC) settings, a meta-analysis reviewing its prevalence in more than ten countries showed that the overall prevalence of depression was 19.5% [3]. In the Spanish population, prevalence rates of 29.0% have been observed for major depressive disorder and 14.6% for dysthymia in PC services [4].

Although previous studies have reported that a combination of psychotherapy and pharmacotherapy is the best treatment option [5], the usual intervention in PC settings is pharmacotherapy alone, even though it produces high relapse rates [6, 7] that increase with each depressive episode [8]. Some patients prefer psychotherapy to pharmacological treatment [9], but it should be noted that Spanish specialized services are overloaded. Moreover, it is difficult to introduce psychotherapy in PC due to problems such as the professionals’ training, high costs or work overload, professionals’ attitudes and organization, geographical and logistic difficulties, and, especially, lack of time [10]. All these problems reveal the need to offer new forms of treatment for depression in PC, and low-intensity interventions seem to be a promising solution.

On the one hand, interventions that are considered low intensity are either not guided (e.g. self-help) by highly qualified facilitators, or they are guided by highly qualified facilitators for only a short time [11]. On the other hand, brief interventions have been operationalized as consisting of more than two and less than ten appointments [12]. Although brief psychotherapies for depression are an alternative that has already been shown to be effective [13], a systematic review of low-intensity psychological interventions for depression revealed the need for more studies [9]. Mindfulness-based interventions (MBIs) have demonstrated their effectiveness in the treatment and prevention of relapse or recurrence of depression. Specifically, Mindfulness-Based Cognitive Therapy (MBCT), an intervention that combines mindfulness training and elements of cognitive therapy [14], has shown significant effects in patients with diagnostic levels of depression. In fact, NICE guidelines recommend it as the treatment of choice for depression recurrence [15]. Although there is no agreement on this topic, many authors believe that MBIs included under the umbrella of “third wave” cognitive behavioral therapy can be considered low intensity interventions. Some studies have demonstrated beneficial effects of brief mindfulness-based interventions in non-clinical populations [16–19].

In addition, internet-based treatments have been found to be promising for numerous mental health problems, including depressive symptoms [20], and for increasing the dissemination of evidence-based treatments [21]. The main advantages of these types of programs are that they reduce the contact time between the patient and the clinician, and they reach patients who would not otherwise receive treatment or are geographically distant. In addition, receiving treatment at home ensures confidentiality, minimizes stigma, and reduces the difficulty some people have when receiving psychological treatment [21]. Several systematic reviews have shown that Internet-based treatments based on Cognitive Behavioral Therapy (CBT) are effective, acceptable, and practical for the treatment of health problems such as anxiety and depressive disorders [22–25]. Furthermore, even self-guided online psychological treatments for depressive symptoms have been found to produce higher effect sizes compared to control groups [22], and in general, online treatments seem to be as efficacious as face-to-face treatment as usual for depression [5, 26, 27]. In the case of mindfulness, some studies have applied MBIs over the Internet with good results [28–30], but a literature review and meta-analysis have shown that, although online MBIs have significant effects on improving mental health, these effects could be small to moderate. Moreover, both studies concluded that more research is needed using more rigorous methods and examining long-term effects and moderators of online MBIs [27, 31].

Therefore, due to the need for further research about the possible benefits of low intensity interventions in PC settings and brief MBI applications, this study aims to compare a low-intensity mindfulness intervention for the treatment of depression in PC using different intervention formats – a face-to-face MBI in a group format (6-8 people/group) and the same MBI but individually applied on the Internet – to a control group receiving PC medical treatment as usual (TAU). The cost-effectiveness of the intervention formats, compared to TAU, is also evaluated.

The main hypothesis is that the MBI programs (face-to-face and online) will be more effective than TAU. Furthermore, improvements are also expected in secondary outcome measures (depression, positive and negative affect, and happiness) and other measures (health-related quality of life, mindfulness skills) in both intervention groups, compared to the TAU group. Additionally, the differences between face-to-face and online formats will be explored because, to our knowledge, there are no studies to date that compare MBIs applied online vs. face-to-face in this population. Finally, regarding cost-effectiveness, we expect that both MBI interventions will be more cost-effective than TAU. Additionally, cost-effectiveness differences between face-to-face and online formats will be explored.

## Methods

### Study design

**Fig. 1:**
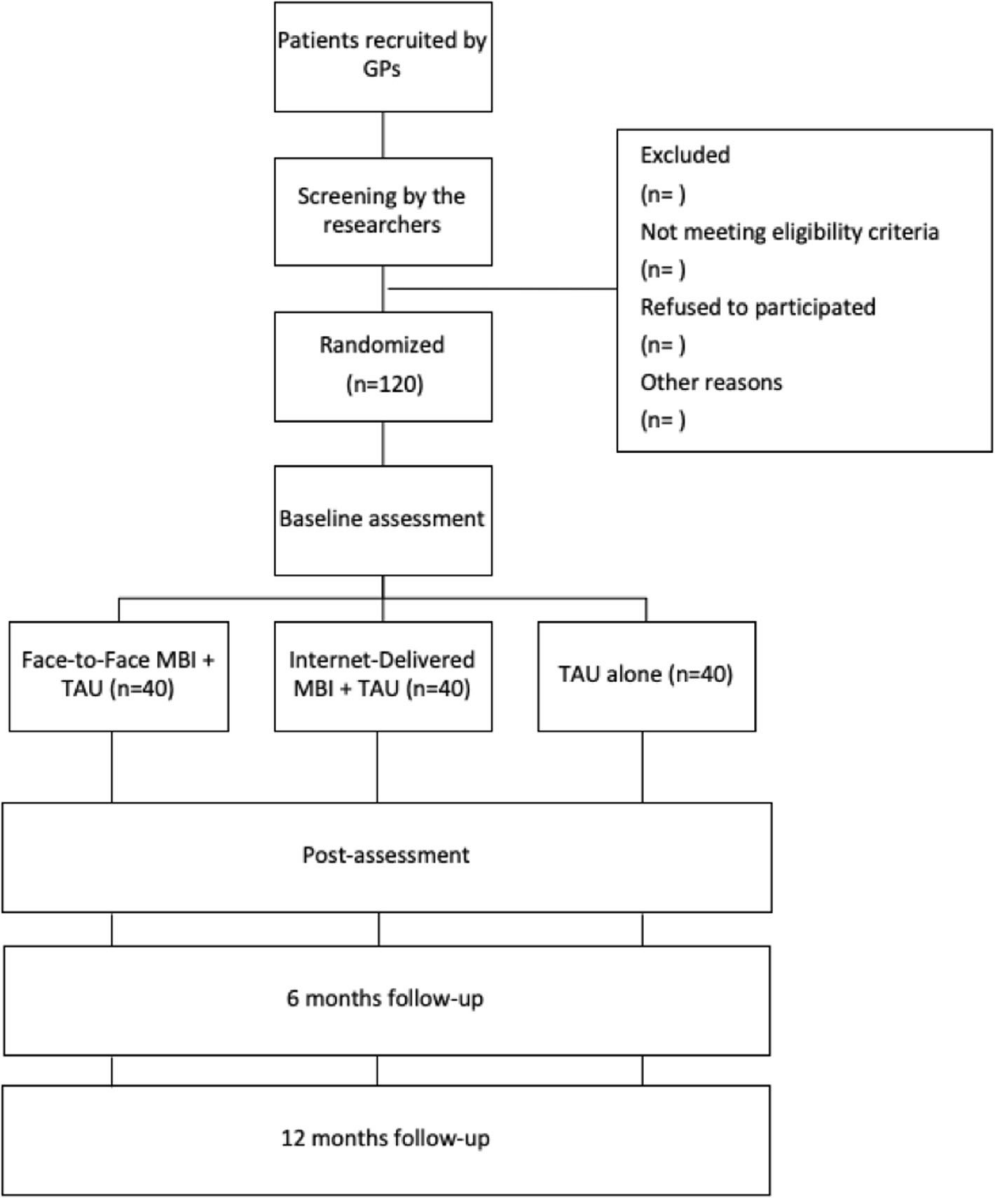
Flowchart of the study

Participants will be randomly allocated to a three-armed randomized controlled clinical trial with three conditions: a) “face-to-face MBI + TAU”, b) “Internet-delivered MBI + TAU”, and c) “TAU alone”. They will be assessed at pre- and post-treatment and 6- and 12-month follow-ups. The study flowchart is shown in Fig. 1. The study is currently in progress in the data recruitment stage.

This study will follow the CONSORT statement (Consolidated Standards of Reporting Trials, http://www.consort-statement.org) [32] and the SPIRIT guidelines (Standard Protocol Items: Recommendations for Intervention Trials) [33]. The study’s trial registration number is *ClinicalTrials.gov* NCT03034343.

### Ethics

This study will be conducted according to the international standards of the Declaration of Helsinki and subsequent amendments. This trial will be performed in compliance with the study protocol and with good clinical practice guidelines with the main aim of protecting and preserving human rights [34]. Data security will be guaranteed, the participants included in the study will be protected by the Organic Law on Protection of Personal Data (15/1999 of December 13, LOPD) and all relevant EU legislation in this regard, and international privacy agreements will be observed and respected. The Internet platform will be accessed through a unique username + password combination. An advanced encryption standard will be used to protect the data (AES-256). The study has been approved by the Ethics Committee of Universitat Jaume I (Castellón, Spain) and the Ethics Committee of Aragón (Zaragoza, Spain). An informed consent form will be signed by participants before randomization. Participants allocated to the TAU arm will also be offered the possibility of receiving the psychotherapy program through the format they prefer with all the contents and materials at the end of the study.

### Study population, recruitment, and eligibility criteria

This randomized controlled clinical trial will be conducted from the Health Center of Arrabal in Zaragoza (Spain). Participants will be adult PC outpatients attended to by their corresponding General Practitioners (GP), and they will be recruited by GPs working in the different Health Centers in Zaragoza (Spain). The researchers who manage the study will go to the different Health Centers to explain the study and eligibility criteria to the GPs, and they will give them an information sheet containing the study characteristics.

When a GP identifies a potential patient, s/he will explain the study to him/her and inform him/her that s/he can refuse to participate or withdraw from the study at any time, without any negative effects on their professional relationship or on future treatments that might be offered to the participant in PC. If the patient is interested, s/he will have to come to the Arrabal Health Center to clarify any doubts about the study and sign an informed consent form. Then, the patient will undergo a clinical interview and fill out the paper-and-pencil screening survey describing his/her sociodemographic and clinical characteristics. This first data collection and screening will be performed by specially trained psychologists with the objective of assessing whether the participants meet the eligibility criteria to participate in the study. If the participant meets the inclusion criteria, an independent researcher will implement randomization by telephone to avoid predictability.

Inclusion criteria will be as follows: a) older than 18 years; b) DSM-5 diagnosis of Major Depression or Dysthymia or mild or moderate depression, expressed as a score between 5 and 14 on the Patient Health Questionnaire (PHQ) [35]; c) depressive symptoms present for at least 2 months; d) having a computer and Internet at home; e) being able to read and understand the Spanish language; and f) willingness to participate in the study and sign the written informed consent form.

Exclusion criteria include: a) any diagnosis of a disease that may affect the central nervous system (brain pathology, traumatic brain injury, or dementia); b) other psychiatric diagnoses or acute psychiatric illnesses (severe range of depression, substance dependence or abuse, history of schizophrenia or other psychotic disorders, and eating disorders), except for anxious pathology or personality disorders; c) any medical, infectious, or degenerative disease that may affect mood, presence of delusional ideas, or hallucinations consistent or not with mood and suicide risk. Participants in both the TAU group and the MBI conditions can continue their medication (mainly anxiolytics and antidepressants) if there are no increases, unless they are necessary. However, they will not be allowed to receive any other psychological treatment during the study period (unless it is necessary for ethical reasons and it benefits the participant). Cases where the medication is increased or another psychological treatment is needed will be excluded from the analysis.

### Randomization and blinding

Participants who meet the inclusion criteria for the study will receive a code from the screening assessors in order to maintain participants’ confidentiality. An independent researcher unaware of the study characteristics will perform the randomization process. In order to randomly allocate the participants to one of the three conditions referred to above (face-to-face MBI + TAU, Internet delivered MBI + TAU, and TAU alone), a computer-generated random number sequence (https://www.randomizer.org/) will be used by means of a simple allocation strategy and a 1:1:1 rate. After informing the patients of the study characteristics, they will accept to participate before the random allocation and without knowing the condition to which they will be allocated. The researcher who administers baseline assessments will be blind to the patients’ treatment group. This researcher will be different from the one – also blinded – who administers the other measures throughout the study. Patients in the Internet-delivered MBI arm will complete all the measures online. GPs will be blind, but the face-to-face MBI therapist will not. Participants will not be blind to the treatment condition for ethical and practical reasons.

### Sample size

The sample size estimation was based on testing whether the trend in the change is different between the intervention groups throughout the 4 time points. Firstly, we assumed that MBI groups would be able to present moderately high effects, compared to “TAU alone”, on the BDI-II at post-treatment. To operationalize this, we considered a standardized difference between arms on the main outcome of d = 0.7, which corresponds to the average effect observed in ten studies comparing MBIs with different wait-list conditions [36] and surpasses the 0.5 standard deviation criterion considered clinically relevant in previous studies [37, 38]. A preceding study by our research group with mild or moderate depressed patients in a similar Spanish PC setting found BDI-II means and standard deviations (SDs) of 22 and 5, respectively [20]. The inclusion criteria used in this study restricted the entry of mild or moderate depressed patients, thus reducing the SD compared to the general population and presenting a less skewed distribution of the data, as we predict will occur in the present study. The effect size referred to above corresponds to a difference of 3.5 points, which implies a reduction of slightly more than 15% in the BDI-II scale used. According to the GLIMMPSE v2.0.0 – time x group interaction in a general linear repeated measures (RM) design – and GPower v3.1.9.4 – ANOVA test for RM within-between interaction – statistical tools, considering the referred difference between the “face-to-face MBI + TAU” vs. “TAU alone” groups of depressed patients, which corresponds to a partial eta square value of roughly 0.09 and an effect size f of 0.31, with a common mean at baseline of 22, a mean at post-test of 16 and 19.5 respectively, and a mean at post-test of 17 for the “Internet delivered MBI + TAU” condition, and assuming a one point reduction in each group at each following time point, a baseline correlation of 0.6 and a decay rate of 0.35, assuming a common SD of 5, a 5% significance level, a statistical power of 80% using a 1:1:1 ratio, and an univariate approach to RM with Greenhouse-Geisser correction [39], we needed 33 subjects in each group. Because we expected a dropout rate of approximately 20% [40], we inflated the numbers to reach a total sample size of 120 patients, 40 per arm [41].

### Interventions

#### Mindfulness-based interventions (MBIs)

The two MBIs (face-to-face and Internet delivered) are composed of an initial face-to-face group (6-8 participants) session to introduce the therapeutic modules, explain how mindfulness can help patients with their problem, and motivate them to change. In the case of online patients, the therapist will also explain how to enter the web page where the therapeutic modules are located. Thus, both conditions are composed of four modules, face-to-face or online, depending on the assigned condition. Regardless of the condition, all the patients will receive their corresponding TAU.

- “*Face-to-face MBI” sessions* will be carried out in a group format (between 6 and 8 participants) and with weekly sessions lasting 90 min each for 4 weeks. The therapist will explain the different modules and how to do the mindfulness exercises. The mindfulness activities will be performed in the sessions, and then participants will have to practice at home. All the materials will be delivered in print form, except the audios, which will be sent by e-mail. However, the audios will also be transcribed in the printed modules. Module contents are the same as those in the online intervention. These contents have been adapted from an online format previously used [10] to a traditional format for their application in this study. If a participant does not attend a session, the corresponding materials will be delivered to him/her, and s/he will be able to ask any questions about them in the next session.

- “*Internet-based MBI”* will be carried out online and individually through a web platform (https://www.psicologiaytecnologia.com/) where participants will have to introduce a login and a password to enter their corresponding profiles. The duration of each module will be approximately 60-90 min, depending on the participant’s pace and time availability, and the estimated program duration for most people will be between 4 and 8 weeks [10]. Some meta-analyses have shown that attrition rates are higher when no therapist support is provided to patients in Internet-based programs [21, 42]. For this reason, when the patients complete the second module (halfway through the program), they will receive a phone call from the therapist who will monitor their progress and motivate them to continue and adhere to the program. However, no additional therapeutic content will be offered.

Before the therapeutic modules, there is an *introductory module* (M0) with basic information about emotional disorders, the protocol contents, the importance of evaluation and registers, and ambivalence as a natural part of change, as well as an exercise where the participant fills in a table with the costs and benefits of the change process. This module is carried out face-to-face in both treatment conditions before the pre-treatment assessment and the intervention, and it is presented individually to the Internet-based MBI arm and in group format to the face-to-face MBI arm.

The therapeutic modules are oriented toward working on different formal psychological mindfulness practices that can help to decrease depressive symptomatology. These practices emphasize thought management, acceptance, values orientation, and compassion over a short period of time. For this reason, a brief intervention is proposed, composed of four modules (a brief description of each module is presented below).

##### M1. Getting to know mindfulness

This module will show what mindfulness is, prejudices about it, inattention problems in our society, some mindfulness benefits, and recommendations for practicing it. In this module, participants will perform the raisin mindfulness exercise, which is widely used as an introduction to mindfulness meaning.

##### M2. Establishing formal and informal practices

This module will explain the importance of formal and informal practice. To do so, different techniques such as the three-minute practice will be demonstrated.

##### M3. Thoughts management, body scan practice, and values

This module aims to show the importance of thoughts and values in our emotions and lives, and the need to recognize them. It will also include the body scan practice.

##### M4. Self-compassion and integrating mindfulness in everyday life

This module works on how to establish a mindfulness practice habit in order to consolidate the use of this technique. It will also include a self-compassion practice, which has shown its efficacy in treating depression symptoms [10, 33].

These modules can be done step-by-step. When a patient has finished a module, it can be reviewed as many times as necessary. Each module includes: content explanations, self-assessment questions to test whether participants have understood the contents, exercises to practice, and homework assignments.

#### Treatment as usual (TAU)

Participants in the treatment as usual (TAU) arm will continue to be treated by their GPs in their corresponding health centers. The treatments offered by the GPs are mainly pharmacological, with antidepressants and/or anxiolytics being the most frequent medications. In cases of suicide risk or other severe symptoms or unexpected effects during the study, the patient will be referred to specialized mental health care services and excluded from the data analysis.

### Instruments

The evaluations will be performed at screening, baseline, post-treatment, and 6- and 12-month follow-ups. All variables and assessment periods of the study can be found in Table 1. Scores on depression, negative and positive affect, and opinions about the modules will also be obtained after each completed module in the face-to-face and Interned-based MBIs. Participants in the TAU group will report their general opinion about the treatment as usual received at the end of the program.

**Table 1:** Study outcomes

Instruments	Assessment Area	Time of assessment^a^
Sociodemographic	Gender, age, marital status, education, occupation, economical level.	Screening
MINI	Psychiatric diagnosis	Screening
PHQ-9	Severity of depression	Screening, post-module, post-treatment and follow-ups
CSRI	Health and social services use	Screening and 12-month follow-up
BDI-II	Severity of depression	Baseline, post-treatment and follow-ups
SF-12	Health-related quality of life	Baseline, post-treatment and follow-ups
EQ-5D	Health-related quality of life	Baseline and follow-ups
PANAS	Positive and negative Affect	Baseline, post-modules and follow-ups
FFMQ	Facets and factors of mindfulness	Baseline and follow-ups
PHI	Remembered and experienced well-being	Baseline and follow-ups
ETS	Treatment expectations	Baseline
OTS	Treatment opinions	Post-treatment

*MINI* Mini-International Neuropsychiatric Interview, *PHQ-9* Patient Health Questionnaire-9, *CSRI* Client Service Receipt Inventory, *BDI-II* Beck Depression Inventory-II, *SF-12* Short Form-12 Health Survey, *EQ-5D* EuroQoL-5D Questionnaire, *PANAS* Positive and Negative Affect Scale, *FFMQ* Five Facet Mindfulness Questionnaire, *PHI* Pemberton Happiness Index, *ETS* Expectation of Treatment Scale, *OTS* Opinion of Treatment Scale.

^a^Waves: screening, baseline, post-treatment (6 weeks for the “face-to-face MBI + TAU” and “TAU alone” or 12 weeks after baseline for the “Internet-delivered MBI + TAU”), post-module (after each individual module for the “face-to-face MBI + TAU” and for the “Internet-delivered MBI + TAU”), follow-ups (both 6 and 12 month follow-up measurements)

#### Diagnosis interview

##### -Mini international neuropsychiatric interview V-5.0.0 (MINI)

The MINI V-5.0.0 interview is structured based on the psychiatric diagnoses of DSM-IV and ICD-10 [43, 44]. To administer it, clinicians only need a brief training and a short period of time that oscillates around 15 min. This interview has proven to be an accurate interview for use in multicenter clinical trials. The MINI interview will be used in the screening to determine whether a potential participant meets the inclusion criteria for Major Depression or Dysthymia. However, for cost reasons, the MINI interview will not be applied at post-treatment or follow-up. The MINI V-5.0.0 has been translated and validated in the Spanish language [31, 35].

#### Primary outcome

##### *-Beck Depression Inventory II (BDI-II)* [45]

The BDI-II total score, as a continuous variable, will be used as the primary outcome measure at post-test. It has been widely used in clinical research to assess changes in depression severity [20]. Additionally, the BDI-II has shown good sensitivity and specificity in detecting major depressive disorder in the Spanish-speaking population [46]. It is composed of 21 items about symptoms characterizing major depression. The BDI-II total score can be obtained by adding up the answers, from a minimum of 0 to a maximum of 63 points. The BDI-II Spanish version has shown high internal consistency in both general (α = 0.87) and clinical populations (α = .89) [47].

#### Secondary outcomes

##### -Sociodemographic variables

The sociodemographic variables that will be collected are the following: gender, age, marital status (single, married/relationship, separated/divorced, and widowed), education (no studies but can read and write, primary education, secondary education, University studies, and others), occupation (student, housework, unemployed, employee, sick leave, retired, disability, and other), and economic level (< 1 minimum inter-professional salary –MIS, 1-2 MIS, 2-4 MIS, and > 4 MIS).

##### *-The Patient Health Questionnaire 9 (PHQ-9)* [48]

This questionnaire is also one of the most widely used in research to evaluate depression severity. It is a brief instrument consisting of 9 items scored from 0 (“not at all”) to 3 (“nearly every day”), and it is completed by the patient. In this trial, the Spanish validated version will be used [35].

##### -Positive and negative affect scale (PANAS) [49]

This questionnaire is composed of 20 items and two independent dimensions: positive affect (PA) and negative effect (NA). Each scale has 10 items, and the score range for each is from 10 to 50. The psychometric properties of the PANAS are satisfactory. The Spanish version will be used [50].

##### *-The Pemberton Happiness Index (PHI)* [51]

The PHI is used to assess remembered well-being (general, hedonic, eudaimonic, and social well-being) through 11 items rated on a Likert scale ranging from 0 (strongly disagree) to 10 (strongly agree). In addition, experienced well-being (positive and negative emotional events that may have happened the day before) is also measured through 10 items answered dichotomously (yes or no). In this study, the Spanish validated version will be used [51].

### Other outcomes

#### Health-related quality of life

##### *-Sort Form-12 Health Survey* (SF-12) [52]

This instrument measures health-related quality of life and general functioning. The SF-12 consists of 12 items that have demonstrated adequate psychometric properties in its Spanish version [53, 54].

##### *-EuroQoL-5D* (EQ-5D) [55, 56]

The EQ-5D is an instrument commonly used to measure health-related quality of life. Participants self-report problems in the following five domains: mobility, self-care, usual activities, pain/discomfort, and anxiety/depression. Each one of them is divided into three severity levels corresponding to no problems, some problems, and extreme problems, which makes it possible to obtain a population-based score or societal index (SI). In addition, the participant will self-assess his/her current health status on a 10 cm vertical line where the best and worst imaginable health states score 100 and 0, respectively. The scores for these health-related states will be assigned using the readily available Spanish population rates [40, 41].

#### Cost-effectiveness

##### *-Client Service Receipt Inventory* (CSRI) [57]

This questionnaire is used to collect information about healthcare uses and social care services, in addition to other economic impacts (such as time off work due to illness). The variant used in this study was designed to collect retrospective data on service utilization during the 6 months prior to the assessment. The Spanish validated version will be used [58].

#### Therapeutic process measure

##### *-Five Facet Mindfulness Questionnaire (FFMQ)* [59]

This questionnaire assesses five factors of mindfulness through 39 items: observing (8 items), describing (8 items), acting with awareness (8 items), not judging inner experience (8 items), and not reacting to inner experience (7 items). Items are answered on a Likert-type scale ranging from 1 (“never or very rarely true”) to 5 (“very often or always true”). The FFMQ has been shown to have good internal consistency and reliability in its Spanish validation [60].

#### Treatment expectations, opinions, and completion

##### -Expectation of treatment scale (ETS) and opinion of treatment scale (OTS)

These scales are adapted from Borkovec and Nau [61]. They include 5 items, rated from 0 (“not at all”) to 10 (“very much”), that address how logical the treatment seems, to what extent it satisfies the patient, whether it could be used to treat other psychological problems, its usefulness for the patient’s specific problem, and to what extent the treatment might be aversive. The expectation scale will be applied once the treatment rationale has been explained and after M0 to measure subjective patient expectations about the treatment they are about to receive. The opinion scale is administered when the patients have completed the treatment to assess their satisfaction with it.

In the case of the MBI groups, a question will be introduced related to the opinion about each individual module (e.g. “To what extent has this module been useful to you?“), using a visual analogue scale ranging from 0 (“nothing”) to 10 (“very much”), as well as two questions related to reviewing the materials (“Have you reviewed the last module seen in the session and all the materials it includes?”) and homework completion (“Have you done the tasks to train at home?”), with two possible responses (yes vs. no). In addition, a module completion register will be filled out.

### Data analysis

The results will be reported following CONSORT recommendations [62, 63]. Socio-demographic and clinical information will be described at baseline by means of frequencies and percentages (e.g. categorical variables) or means and standard deviations (e.g. continuous variables), and treatment conditions will be compared to ensure the success of the randomization, using the chi-square or Fisher tests and one-way ANOVA.

### Main analysis

The efficacy of the “face-to-face MBI + TAU” group compared to the “TAU alone” group will be estimated based on the main BDI-II outcome, which will be considered a continuous variable. Multilevel mixed-effects models will be developed by means of a RM design on an intention-to-treat (ITT) basis, using the restricted maximum likelihood (REML) method, in order to produce unbiased estimates of variance/covariance parameters [54]. Non-standardized slopes and 95% confidence intervals (95% CI) will be calculated by adjusting those variables that show significant differences between groups at baseline. In order to study the specific trajectories of each group throughout the trial and determine whether possible differences between groups are consistent over time, the ‘group x time’ interactions will be calculated. Cohen’s d effect sizes will be calculated at each time point using the combined standard deviation at baseline [64]. Effect sizes are small when d ≤ 0.2; medium when d = 0.5; and large when d ≥ 0.8 [65].

### Secondary analysis

The efficacy of the “face-to-face MBI + TAU” group compared to the “TAU alone” group with regard to the secondary outcomes and the other measures, and the efficacy of the “Internet-delivered MBI + TAU” compared to the “TAU alone”, as well as the exploratory comparison of the “face-to-face MBI + TAU” and “Internet-delivered MBI + TAU” on the primary and secondary outcomes, will be calculated following the same analytical strategy used for the main analysis. Per protocol analysis will also be performed, considering only those patients who attend at least 3 modules (out of 4). The clinical significance of improvements between groups will be explored by calculating the absolute risk reduction and number needed to treat (NNT) (and their 95% CI). We will use two criteria for improvement: a) changing to a less severe cluster in the BDI-II compared to the one the patient was allocated to at baseline; c) calculating the clinical significance of improvements by establishing both the cut-off point and reliable change index on the BDI-II total score using the Jacobson and Truax method [66]. Additionally, possible differences in PHQ-9 and PANAS will be explored throughout the MBI modules using mixed models.

### Cost analysis

Cost will be calculated from healthcare and societal perspectives during the 12 months before the screening and during the 12 months before the last follow-up. The cost of pharmacological therapy, medical tests, and use of health services will be added together to calculate direct healthcare costs. Indirect costs such as loss of productivity will be calculated considering the minimum daily wage in Spain and the number of sickness absence days. Total costs will be estimated by adding up direct and indirect costs in €. The effectiveness of the interventions will be estimated as the difference between the BDI-II score at baseline and at follow-up, and utility will be estimated using quality-adjusted life-year (QALYs) at follow-up. QALYs will be calculated using the area-under-the-curve (AUC). Cost-effectiveness will be analyzed through the estimation of incremental cost-effectiveness ratios (ICERs). Cost-utility will be calculated through the estimation of incremental cost-utility ratios (ICURs). Cost-utility planes will be plotted.

### Level of significance

An alpha level of 0.05 will be established using a two-tailed test. The probability values relative to the main analysis will be adjusted according to Benjamini-Hochberg’s correction for multiple comparisons, but the secondary and exploratory analyses will not be corrected.

## Discussion

Consistent with the evidence, a large percentage of patients seen in PC services are being treated long-term with antidepressants, showing high relapse rates when trying to stop the medication [67], and many of them are referred to already overloaded specialized mental health services [68]. Therefore, there is a need to offer new forms of treatment that provide stability or improvement before moving on to specialized care. Currently, a growing number of studies show the efficacy of MBIs for emotional disorders, but only a few have focused on PC settings [69]. In this context, the present study protocol describes a randomized controlled clinical trial that has the main objective of evaluating a low-intensity MBI plus TAU for the treatment of depression in PC services compared to TAU alone.

Additionally, the advantages of brief Internet-delivered psychotherapy for depression could provide an alternative to current treatments and a possible solution for existing problems, especially with regard to waiting lists, lack of time, and health costs of PC Spanish services. Moreover, the effectiveness of Internet-based treatments for this disorder has also been demonstrated, and this kind of protocol can help to spread evidence-based treatments, so that more people can benefit from them [21, 70]. Specifically, mindfulness has been proposed as a potential non-drug therapy for mild-to-moderate depression [71] and for preventing depression relapses [72]. The study of mindfulness-based online interventions is increasing due to their good results and the benefits that have begun to be observed [27, 31].

The study has various strengths. First, to the best of our knowledge, this is the first Spanish randomized controlled clinical trial to apply a face-to-face brief MBI, and the second study to apply an online low-intensity MBI in PC settings [10]. Second, this study proposes two different ways to apply mindfulness treatments, which will allow us to compare them with TAU and find out which one is more cost-effective. Lastly, this will be the first study to compare a traditional face-to-face vs. online mindfulness program in the Spanish PC context, although in an exploratory way.

The limitations are similar to those of the study carried out by Castro et al. [10], who refer to possible negative attitudes from GPs about recommending this treatment, and high dropout rates in the treatment groups, especially in the control group. To minimize the effect of negative attitudes, the intervention characteristics will be explained in depth to the clinicians involved in the trial. In order to prevent dropouts, we will send emails to the participants reminding them to continue and complete the trial requirements. Another limitation is the possible small statistical power to obtain significant results in the comparison of the face-to-face and online MBI groups, but, as mentioned above, this study will be considered a pilot exploration of possible effect sizes to guide future research. Other difficulties that may appear could be related to recruitment because some people might not have access to the Internet at home, or they might even prefer traditional treatments (due to unawareness of the benefits of the new treatments and technologies) or be influenced by the depressive symptoms themselves (apathy, anhedonia, etc.).

### Clinical implications

Positive results of this randomized controlled clinical trial may have an important impact on public PC and mental health settings because applying a brief psychotherapeutic protocol to treat mild or moderate depression could help to decrease the overload of the public mental health system. On the one hand, it could reduce costs, waiting lists, hours of clinical assistance, and hours of face-to-face treatment. On the other hand, it offers treatment alternatives beyond antidepressant medication that can help to reduce depressive symptoms and slow down the inclusion of patients in mental health services by offering high-quality and flexible interventions in PC [73].

Finally, benefits of mindfulness in different disorders and in quality of life outcomes have been reported by several studies [74, 75]. Thus, although this study focuses mainly on depression, this proposal could also be used as a trans-diagnostic protocol in other comorbid psychological problems or emotional disorders (e.g. anxiety disorders) [71]. However, this is a brief treatment protocol, and research should be carried out in this regard. In conclusion, significant results from this trial could be beneficial for society and public health as a whole and encourage further research along these lines.

## Data Availability

Not applicable.

## Abbreviations

AUC: Area-under-the-curve
BDI-II: Beck depression inventory II
CBT: Cognitive behavioral therapy
CI: Confidence intervals
CSRI: Client service receipt inventory
EQ-5D: EuroQoL-5D
ETS: Expectation of treatment scale
FFMQ: Five facet mindfulness questionnaire
GP: General practitioner
ICERs: Cost-effectiveness ratios
ICURs: Cost-utility ratios
ITT: Intention to treat
MBCT: Mindfulness-based cognitive therapy
MINI: Mini international neuropsychiatric interview V-5.0.0
OTS: Opinion of treatment scale
PANAS: Positive and negative affect scale
PC: Primary care
PHI: Pemberton happiness index
PHQ: Patient health questionnaire
QALYs: Quality-adjusted life-year
REML: Maximum likelihood
RM: Repeated measures
SF-12: Sort Form-12 health survey
TAU: Treatment as usual
WHO: World health organization
